# A Body Shape Index and Heart Rate Variability in Healthy Indians with Low Body Mass Index

**DOI:** 10.1155/2014/865313

**Published:** 2014-10-02

**Authors:** Sharma Sowmya, Tinku Thomas, Ankalmadagu Venkatsubbareddy Bharathi, Sambashivaiah Sucharita

**Affiliations:** ^1^Department of Physiology, St. John's Medical College, Bangalore 560034, India; ^2^Division of Epidemiology and Statistics, St. John's Research Institute, Bangalore 560034, India; ^3^Clinical Physiology Unit, Department of Physiology, St. John's Medical College, Bangalore 560034, India

## Abstract

*Background.* One third of Indian population is said to be suffering from chronic energy deficiency (CED), with increased risk of developing chronic diseases. A new anthropometric measure called A Body Shape Index (ABSI) is said to be a better index in predicting risks for premature mortality. ABSI is also in part said to be a surrogate of visceral fat. *Objective*. The present study aimed to explore the association between indices of HRV (heart rate variability), BMI, WC, and ABSI in healthy Indian males with low BMI (BMI < 18.5 kg/m^2^) and to compare with normal BMI group (BMI 18.5 to 24.9 kg/m^2^). *Methodology*. ABSI and BMI were derived from anthropometric parameters, namely, height, weight, and waist circumference in 178 males aged 18 to 78 years. Subjects were categorized into two groups based on their BMI. *Results and Conclusions*. Power spectral analysis of HRV demonstrated a significant negative correlation between Log HF (high frequency) and ABSI in both low BMI [−24.2 (9.4), *P* < 0.05] and normal BMI group [−23.41 (10.1), *P* < 0.05] even after controlling for age. Thus even with slight increase in BMI among low BMI individuals, there could be a greater risk of cardiovascular morbidity and mortality.

## 1. Introduction

Obesity has been the center of focus of majority of body composition related studies. This is partly due to a large body of data available from western population where obesity is considered a major public health concern [1–3]. Epidemiological studies with longitudinal follow-up have demonstrated association between simple anthropometric measures like height, weight (body mass index: BMI), and waist circumference (WC) with cardiovascular risks [3, 4]. Within a given BMI range, association with increased cardiovascular risks becomes even stronger as WC increases [5]. Studies have linked alterations in autonomic nervous activity with increased cardiovascular risk [6]. Heart rate variability (HRV) is a simple noninvasive, sensitive measure to evaluate autonomic system activity. Changes in HRV have been demonstrated to detect even subclinical states [7, 8]. Decreased heart rate variability (both low and high frequency) has been observed in obese, compared to individuals with normal BMI [9–11]. Pockets of data available from Indian population are not different from those of western data [12, 13]. However, India has an additional burden of chronic energy deficiency with one-third of population being of low BMI [14]. Recent evidence based on HRV measures from our laboratory demonstrated decreased HRV in undernourished subjects when compared to well-nourished/normal BMI group [15]. It has been speculated that undernourished/low BMI individuals might have more of visceral adiposity or ectopic fat than subcutaneous fat [16]. Although WC has been suggested as being superior to BMI in predicting cardiovascular risks in obese [17], its ability to predict increased cardiovascular risk in individuals with low BMI needs to be explored. Further, WC is highly sensitive to body size and fat distribution and is correlated with BMI. Thus, differentiating BMI and WC as two independent epidemiological risk factors becomes difficult. Recently a new anthropometric measure, called A Body Shape Index (ABSI), has been derived from waist circumference which is independent of BMI and is said to be a better index than using either WC or BMI independently. Thus being independent of BMI, ABSI helps in understanding whether abdominal obesity has predictive ability that cannot be explained by BMI alone. Current study aimed to explore the association between indices of HRV and the anthropometric measures including BMI, WC, and ABSI in healthy Indians with low BMI and compare it with normal.

## 2. Materials and Methods

One hundred and seventy eight males aged 18 to 78 years were recruited from in and around the medical college. Based on their BMI, subjects were categorized into low BMI group (<18.5 kg/m^2^) and normal BMI group (between 18.5 and 24.9 kg/m^2^). Subjects with any chronic ailments including diabetes, hypertension, and renal and endocrinal disorders were excluded. None of the subjects reported any symptoms suggestive of peripheral or autonomic neuropathy like giddiness on standing, urinary urgency, tingling sensation of limbs, or limb weakness or gastrointestinal symptoms like burning sensation in epigastric region, diarrhea, and constipation. The institutional ethical review board approved the research protocol.

All subjects underwent anthropometric assessment including weight recorded in minimal clothing to the nearest 0.1 kg, using a digital scale (Alfoset, Model HW-100KA1, Mumbai, India) and height being recorded to the nearest 0.1 centimeter, using a vertically mobile scale (Holtain, Crymych, UK). Body mass index was calculated as weight/height^2^. WC was measured using a standard nonstretchable tape measure, at the narrowest point between the iliac crest and ribcage. The measured WC from our population was compared with the predicted WC derived from the equation log⁡(WC) = (−2.589 ± 0.020)+(0.6807 ± 0.0052)log⁡  weight − (0.814 ± 0.020)log⁡  height [18]. The measured WC was used for subsequent analysis. ABSI was defined as WC/(BMI)^2/3^ ×  (Height)^1/2^. In the above equation, weight was expressed in kilograms and height and WC in meters [18].

### 2.1. Heart Rate Variability Assessment

#### 2.1.1. Preparation of the Subject

After explaining the process of ECG recording, subjects were asked to rest and relax in supine posture for 30 minutes. ECG recording was performed in a quiet room with soothing lighting and with comfortable temperature being maintained. Any electronic gadgets or metal or magnetic objects which could interfere with the ECG recording were removed. Skin was kept dry and rubbed with alcohol pad before application of electrodes.

#### 2.1.2. Electrode Placement

Disposable electrodes were placed firmly onto the skin and we made sure that a good contact between the skin and the electrode was maintained. Electrodes were placed on the right arm, left arm, and left leg (lead II).

#### 2.1.3. ECG Module

ECG module contains the electronics that detect electrical impulses from the heart and convert them into digital data that is transmitted to the computer. One end of the ECG module was connected to the subject through leads and another end to the USB port of the computer.

### 2.2. Processing of the ECG Signals

Analog lead II ECG signal obtained was digitized using analog to digital converter (CIO-AD16Jr A/D card). Sampling frequency of ECG signals was 1000 Hz using an IBM compatible PC and a data acquisition package (CVMS; World Precision Instruments Inc., Sarasota, FL, USA.) The data acquisition system included a threshold peak detection system, from which RR intervals were determined. The RR intervals were plotted as a tachogram (RR values versus interval number). Data segments of 128 sec duration were sampled at 2 Hz to create 256 point data sets. For each 10 min recording, an average of eight data sets of 256 points overlapping by half were processed. Heart rate variability analysis was performed using the frequency domain method. Power spectral density (PSD) was calculated using mathematical algorithm fast Fourier transform (FFT). The PSD was analyzed by calculating powers and peak frequencies for different frequency bands. No more than 3 data points were edited in a given segment [19].

#### 2.2.1. Spectrum Estimation

FFT algorithm uses the signals in the time domain (RR interval) and transforms them into discrete frequency domain representation (power spectra). To prevent digital leakage during FFT Hanning digital filtering was used. The spectra obtained for the different data sets were averaged to reduce variance and to sharpen reproducible central peaks. Power was calculated in three bands. The 0.04–0.15 Hz band of RR power (referred to as the low-frequency band) reflects, at least in part, sympathetic nerve activity to the heart and partly parasympathetic activity, while the 0.15–0.4 Hz band (high-frequency band) reflects parasympathetic nerve activity to the heart. Absolute total power (TP) represents the total variance of the power spectral density, that is, 0–0.4 Hz band (msec^2^). In addition to the absolute power, data for heart rate variability are also presented as normalized units, as recommended [20], where the power in the low- and high-frequency bands are expressed as percentage of the total power minus the power of the very-low-frequency band (0.0–0.04 Hz).

### 2.3. Statistical Analysis

The data was checked for normality using the Kolmogorov-Smirnov test. All normally distributed data are expressed as mean ± SD and median (Quartile1 and Quartile3), when not normally distributed. The differences between low and normal BMI study group were compared using independent *t* test. Bland-Altman plots [21] were constructed to examine limits of agreement between measured WC and the predicted WC. Heart rate variability indices were represented as log transformed (absolute units) data. Correlation of log transformed HRV indices with BMI, WC, and ABSI was examined using Pearson's correlation coefficient. Further, correlation of HRV indices with BMI, WC, and ABSI was examined in low BMI and normal BMI study groups. The correlation of HRV with age, ABSI, and WC was examined using linear regression models in the two BMI groups. Statistical significance was considered at *P* < 0.05. All statistical analyses were performed using SPSS v18, SPSS, Chicago, IL.

## 3. Results

**Figure 1:**
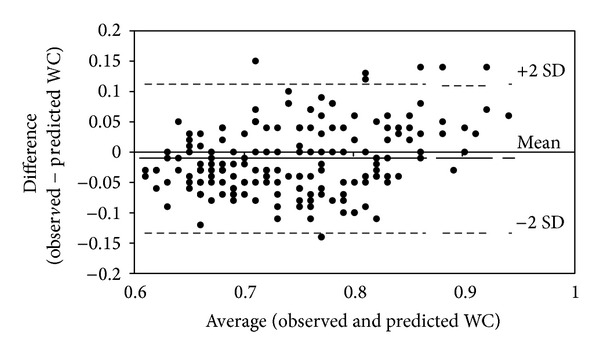
Bland Altman plot of WC differences between observed and predicted values. The solid line is at the bias and the dashed lines are at ±95% limits of agreement.

**Figure 2:**
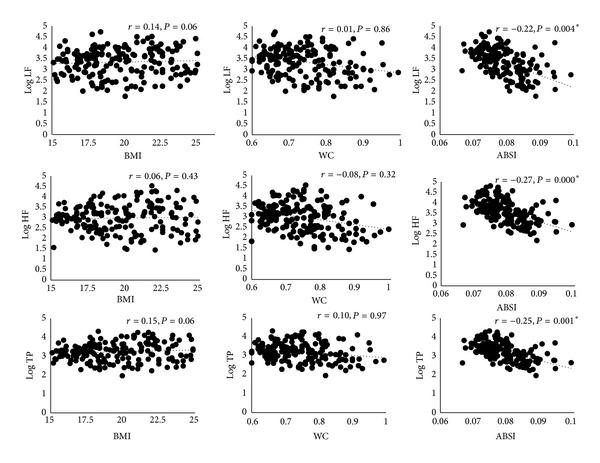
Scatter plots representing the partial correlation between BMI, WC, ABSI, and HRV after adjusting for age. Variables are represented after adjusting for age. *P < 0.05 is considered statistically significant. Correlations without adjusting for age are represented in the results.

Table 1 represents the anthropometric and HRV indices in absolute units. Out of the 178 subjects, based on their BMI, 73 subjects were categorized as low BMI group and 105 subjects as normal BMI group. Predicted WC was within limits of agreement when compared with measured WC. Mean of difference between observed WC from the study population and predicted WC was −0.01 and the limits of agreement were 0.10 and −0.12 (Figure 1). BMI was positively and significantly correlated with WC (*r* = 0.77, *P* < 0.001) and not with ABSI (*r* = 0.028, *P* = 0.71). HRV indices were significantly and negatively correlated with WC (log LF, *r* = −0.25, *P* < 0.01; log HF, *r* = −0.30, *P* < 0.01; log TP, *r* = −0.22, *P* < 0.01) and ABSI (log LF, *r* = −0.54, *P* < 0.01; log HF, *r* = −0.59, *P* < 0.01; log TP, *r* = −0.55, *P* < 0.01) but not with BMI (log LF, *r* = 0.06, *P* = 0.4; log HF, *r* = 0.03, *P* = 0.7; log TP, *r* = 0.1, *P* = 0.2). The correlation of ABSI with HRV remained significant even after controlling for age but not that of WC with HRV (Figure 2). When this correlation was examined separately within low BMI and normal BMI groups, ABSI was significantly correlated with all HRV indices in the normal group after controlling for age, whereas Log HF alone was correlated with ABSI in the low BMI group (Table 2). None of the HRV indices were correlated with WC in either of the groups after controlling for age. There was no significant correlation between the normalized values (nu of LF and HF) and ABSI, BMI or WC.

## 4. Discussion

Current study demonstrated that ABSI was significantly and negatively correlated with LF, HF, and TP indices of HRV. When grouped based on BMI, HF (an indicator of cardiac PNS activity) was negatively and significantly correlated with ABSI in both low BMI and normal BMI study group. However, LF (by far an indicator of cardiac SNS activity) and TP (total variability) were negatively correlated with ABSI only in normal BMI study group.

Nutritional transition is one of the major concerns in India [22]. Increased migration of individuals from rural to urban setup and increased urbanization has caused easy access to high caloric foods [23]. This has caused individuals with low BMI to shift towards either becoming normal BMI or even obese [24]. With reduced physical activity and increased caloric consumption with preexisting low muscle mass, susceptibility to develop chronic disease is increasing in Indians [22–24]. One of the contributing factors is increased body fat accumulation, particularly fat within tissues, which is commonly termed as ectopic/visceral fat. The visceral deposition of fat is considered to be critical in defining the risk for metabolic disease [25] and is relatively more pronounced in Asian Indians compared to other ethnic groups [26]. Visceral fat carries greater risk of insulin resistance, CVS disease, metabolic syndrome [27, 28].

Measures of visceral fat are difficult, tedious and require high-end instruments [29]. Although these techniques might help us give accurate measures, their feasibility and application at a population level are questionable. Simple indices like WC have long been used as a surrogate of central adiposity [30]. Using WC, a simple index called ABSI has emerged of late, which could by far act as a surrogate of visceral fat [18]. In this study there was a good agreement between measured and predicted WC. This allowed us to utilize WC in deriving ABSI for Indian population.

Increasing central adiposity (measured using waist circumference) has been associated with a reduced autonomic functionality [17]. This has been demonstrated in individuals who are obese [31] or who are on a weight loss program [32]. There is lack of understanding of association between indices of central or visceral adiposity and autonomic nervous activity particularly in individuals with low-normal BMI.

Reduced autonomic function predominantly parasympathetic nervous activity has been linked to visceral fat [33]. Christou et al. reported higher abdominal-to-peripheral body fat distributions measured by DXA were strongly correlated with lower sympathetic and parasympathetic function in young and old healthy men [34]. Other studies have also shown that body fat distribution is a major factor, particularly visceral fat carrying the greatest risk for cardiovascular morbidity and mortality [26, 27, 35]. In keeping with this, data from the current study demonstrated that cardiac autonomic activity particularly HF (cardiac parasympathetic activity) was negatively correlated with ABSI across BMI range. This suggests that one of the possible explanations for reduced cardiac parasympathetic activity could well be due to increased ABSI which is by far an indicator of visceral fat.

Greater degree of autonomic involvement, that is, both cardiac sympathetic and parasympathetic nervous activity, is suggested to be associated with higher visceral adiposity [10, 34]. The fact that normal BMI group were having significantly greater WC and marginally higher ABSI though not significant suggests the possibility of greater visceral adiposity in this group. In addition, data from the current study demonstrated a negative association with cardiac sympathetic activity and ABSI in the normal study group. One of the mechanisms which has been linked to greater autonomic involvement (both components of ANS) and visceral fat includes reduced arterial distensibility [36]. Carotid artery distensibility is an important physiological determinant of cardiovagal baroreflex gain [37]. Therefore, it is possible that lower levels of carotid artery distensibility with elevated visceral fat would reduce mechanical transduction of arterial blood pressure into barosensory stretch [33]. Another mechanism could be greater circulating neurohumoural factors that act centrally modifying the cardiovagal baroreflex [38, 39]. Finally, impaired muscarinic receptor function has been demonstrated in an animal model of diet-induced obesity [40]. Thus it is possible that the reduction in cardiovagal baroreflex gain observed with elevated total body and abdominal visceral fat in the present study was due, in part, to reduced cardiac muscarinic receptor number and/or sensitivity.

Thin-fat phenotype, a term coined for Indian population with low-normal BMI, might well have greater visceral adiposity. This calls for urgent need to explore the visceral adiposity status in Indians across all BMI ranges. Possible causes for greater deposition of fat at tissue level may well be due to states of positive energy balance, environmental exposures such as inflammation, stress, tobacco, alcohol, and pollution interacting, and amplifying the effect of the deposition of fat on a thin frame [24]. Studies are further required to explore the role of above factors in contributing towards the deposition of visceral adiposity. Further, studies are also required to explore implication of ABSI in larger population based follow-up studies including females to understand the role of ABSI in predicting the metabolic risk in low BMI group.

In conclusion, greater ABSI by far an indicator of visceral adiposity was associated with reduced cardiac parasympathetic and sympathetic activity even in individuals with low BMI. Thus, even with slight increase in BMI among low BMI individuals, there could be a greater risk of cardiovascular morbidity and mortality.

## Figures and Tables

**Table 1:** Descriptive characteristics of study population.

Subject characteristics	Entire group (*n* = 178)	Normal BMI group (*n* = 105)	Low BMI group (*n* = 73)
Age (Yr)	40 ± 22	41 ± 23	35 ± 22
Weight (Kg)	54.3 ± 9.1	59.3 ± 7.9	47.1 ± 4.9
Height (m)	1.66 ± 0.07	1.66 ± 0.08	1.65 ± 0.07
Waist circumference (m)	0.74 ± 0.08	0.79 ± 0.01	0.68 ± 0.05*
BMI (Kg/m^2^)	19.6 ± 2.6	21.4 ± 1.75	17.08 ± 0.9*
ABSI	0.079 ± 0.006	0.079 ± 0.006	0.078 ± 0.006
Heart rate (bpm)	63.1 ± 9.9	63.8 ± 9.7	62.1 ± 10.3
Low frequency power (LF) (ms^2^)	568.6 (192.7–1155.9)	569.1 (196.1–1405.5)	562.6 (178.3–925.5)
High frequency power (HF) (ms^2^)	538.4 (175.7–1309.4)	572.8 (146.3–1396.1)	522.8 (220.7–872.1)
Total power (TP) (ms^2^)	1843.9 (718.6–3426.9)	2055.9 (685.1–4418.8)	1617.6 (90.3–2935)
Low frequency (nu)	55.2 (38.7–69.0)	59.5 (43.1–74.9)	51.1 (34.7–63.3)
High frequency (nu)	52.9 (40.7–66.2)	49.4 (36.2–63.6)	55.5 (44.9–70.7)
LF nu/HF nu ratio	1.1 (0.6–1.6)	1.2 (0.7–1.9)	0.9 (0.5–1.5)

Grouping based on BMI ranges ≥ 18.5 and < 25 as normal BMI group and BMI < 18.5 as low BMI group.

Data are mean ± standard deviation/median (interquartile range), *P < 0.05.

**Table 2:** Linear regression models describing the associations between HRV indices versus ABSI and WC in normal BMI and low BMI study groups.

Linear regression			Log LF		Log HF		Log TP	
			*β* (Se*β*)		*β* (Se*β*)		*β* (Se*β*)	
			Low BMI	Normal BMI	Low BMI	Normal BMI	Low BMI	Normal BMI
ABSI	Model 1	ABSI	−43.15 (8.5)∗	−46.47 (7.1)∗	−47.51 (7.7)∗	−63.93 (8.6)∗	−37.33 (7.3)∗	−48.61 (6.9)∗
	Model 2	ABSI	−13.73 (10.1)	−21.73 (8.9)∗	−24.20 (9.4)∗	−23.41 (10.1)∗	−15.60 ( 8.9)	−20.33 (8.4)∗
		AGE	−0.01 (0.0)∗	−0.01 (0.0)∗	−0.01 (0.0)∗	0.02 (0.0)∗	−0.01 (0.0)∗	−0.01 (0.0)∗

WC	Model 1	WC	−0.36 (0.0)∗	−0.02 (0.0)∗	−0.04 (0.0)∗	−0.03 (0.0)∗	−0.03 (0.0)∗	−0.02 (0.0)∗
	Model 2	WC	−0.01 (0.0)	−0.01 (0.0)	−0.02 (0.0)	−0.01 (0.0)	−0.01 (0.0)	−0.01 (0.0)
		AGE	−0.01 (0.0)∗	−0.01 (0.0)∗	−0.01 (0.0)∗	−0.02 (0.0)∗	−0.01 (0.0)∗	−0.01 (0.0)∗

Model 1 represents either ABSI or WC. Model 2 represents either ABSI or WC after controlling for age; values represent regression coefficient *β* (standard error of *β*) *P < 0.05.
